# Combination Immunotherapy in Prostate Cancer

**DOI:** 10.3390/cancers14246040

**Published:** 2022-12-08

**Authors:** Constantin N. Baxevanis, Maria Goulielmaki, Angelos D. Gritzapis, Sotirios P. Fortis

**Affiliations:** Cancer Immunology and Immunotherapy Center, Cancer Research Center, Saint Savas Cancer Hospital, 11522 Athens, Greece

## 1. Introduction

During the last decade, there has been significant progress in the field of prostate cancer therapeutic treatments based on androgen receptor-axis-targeted therapies, which resulted in improved clinical outcomes. Notwithstanding this progress, recurrences frequently occur, mostly because such therapeutic modalities do not modulate patients’ immune systems to generate long-lasting, tumor-specific immune memory, which is of paramount importance for inducing durable clinical responses. This consideration highlights the importance of immunotherapy in the field of prostate cancer therapeutics. However, prostate cancer is characterized by low tumor mutational burden (TMB), weak programmed death-ligand 1 (PD-L1) expression, and low frequencies of intratumoral T cells, all of which provide obstacles to effective immunotherapeutic treatments [1]. Nevertheless, there are subgroups of prostate cancer patients whose tumors display immunogenic profiles with increased PD-L1, high TMB, or microsatellite instability and mismatch repair deficiencies, providing superb responses to immunotherapies [2,3]. It is therefore conceivable to determine predictive biomarkers that could assist in selecting prostate cancer patients most likely to respond to immunotherapies. Given the capacity of therapeutic cancer vaccines to generate rigorous antitumor cellular immunity along with immune memory, applying vaccination regimens in the appropriate clinical setting could be advantageous, as, for example, in patients with low tumor burdens having not yet generalized tumor-induced suppression (i.e., patients with recurrent localized disease) [4]. Immune checkpoint inhibitors are not widely explored in terms of function and timing in prostate cancer. Nonetheless, they have been demonstrated to improve the efficacy of therapeutic vaccines when administered in a combined fashion [5,6,7]. The combination of immunotherapy with chemotherapy is also an option for generating antitumor immune reactivity and clinical efficacy, given that chemotherapeutic drugs (i) may circumvent tumor-induced immune suppression by counteracting immune suppressor circuits, and (ii) may stimulate robust antitumor immunity by inducing immunogenic cell death [8,9].

The immune system is positively impacted by androgen deprivation. Thus, by inhibiting either androgen production or androgen-mediating signaling, anti-androgens have been demonstrated to possess immunomodulatory functions, which supports their combined application with immunotherapies [2,10]. Despite the variety of immune-based combination treatments for prostate cancer therapy, there are still important aspects that need to be precisely addressed to maximize their clinical effects. These include, but are not restricted to, (i) the selection of the most effective chemotherapy or hormonal therapy to be administered along with immunotherapies; (ii) the sequencing of administration; and (iii) the capacity of the combined treatments to select for clonal tumor variants, thereby decreasing tumor heterogeneity and achieving significant and long-lasting remissions.

## 2. The Study by Fay EK and Graff JN, Published in Cancers

In their review article, Fay EK and Graff JN [11] underline the essential role of immunotherapy as one of the most important future directions for the therapeutic treatment of prostate cancer. They emphasize the fact that the main achievement of most approaches to cancer immunotherapy is to activate T lymphocytes specifically binding tumor antigen-derived peptides, which will recognize and lyse tumor cells upon migration to the tumor. However, the highly suppressive tumor microenvironment, along with the low frequencies of intratumoral T lymphocytes, hamper the effectiveness of immunotherapies in prostate cancer. Nevertheless, there is a small percentage of prostate cancer patients who have increased TMB and PD-L1 expression responding to immunotherapies. The authors discuss various studies in which immunotherapy has been explored in prostate cancer as monotherapy and in combination with other immunotherapies, chemotherapies, and hormonal therapies. They discuss data on clinical outcomes which clearly show that, although the majority of the patients do not exert meaningful responses, there is still an appreciable percentage of patients exerting significant clinical benefits. This finding is of particular importance as it stimulates studies for the discovery of biomarkers predicting responses to immunotherapies for prostate cancer that are urgently needed for the selection of responding patients. To this end, the authors present six ongoing phase III clinical studies that will evaluate the therapeutic efficacy of immune checkpoint inhibitors (anti-PD-1) in combination with chemotherapy, hormonal therapy, targeted therapy, or anti-CTLA4.

The review is written in an elegant way to highlight the important aspects of prostate cancer immunotherapy with emphasis placed on mechanisms that may hamper clinical responses. The authors take advantage of these suppressive mechanisms to explore combinatorial treatments that could reverse immune suppression and could lead to the discovery of biomarkers that might aid in the prediction of patient responses to immunotherapy. To this end, through this review, the authors make clear that immunotherapy remains an appreciable therapeutic option to control the progression of prostate cancer. Therefore, through immunotherapy-based clinical trials, it will be important to elucidate how to maximize T lymphocyte activation and at the same time diminish intratumoral immunosuppression. These are the two most vital aspects dictating the success of immunotherapy. Tumor-induced immunosuppression provides an obstacle to proper T lymphocyte activation during immunotherapy. However, along with neutralizing immunosuppressive pathways, T lymphocyte activation with subsequent tumor killing should also be positively regulated. For this, combinatorial treatments are needed to target different stages of the dynamic and continuous interactions between tumor cells and immune lymphocytes. Addressing immunosuppressive pathways in conjunction with antitumor T lymphocyte immunity during the progression of prostate cancer should therefore help to develop combination treatment modalities that act in synergy to improve clinical responses. Such combinatorial treatments should progress via immune checkpoint blockade, with any of the standard therapies, including radiation therapy, anti-androgens, and conventional chemotherapy, concurrent with personalized vaccinations. As prostate cancer progresses, it acquires various mechanisms to evade immune surveillance. These involve multi-stage cellular interplays in which cancer cells polarize various components of the tumor stroma to develop a suppressive tumor microenvironment. Moreover, during prostate cancer evolution, tumor heterogeneity develops based on somatic mutations and epigenetic changes which modify various signaling pathways, thus conferring resistance to treatments.

In conclusion, the therapeutic targeting of prostate cancer in its early stages to avoid high levels of tumor heterogeneity is crucial for increasing the possibility of inducing robust clinical efficacy. In addition, a better understanding of the mechanisms underlying responsiveness to immunomodulatory schedules will add to improvements in clinical responses via the design of novel therapeutic strategies.

## References

[1] Mukherjee A.G., Wanjari U.R., Prabakaran D.S., Ganesan R., Renu K., Dey A., Vellingiri B., Kandasamy S., Ramesh T., Gopalakrishnan A.V. (2022). The Cellular and Molecular Immunotherapy in Prostate Cancer. Vaccines.

[2] Bilusic M., Madan R.A., Gulley J.L. (2017). Immunotherapy of Prostate Cancer: Facts and Hopes. Clin. Cancer Res..

[3] Markowski M.C., Shenderov E., Eisenberger M.A., Kachhap S., Pardoll D.M., Denmeade S.R., Antonarakis E.S. (2020). Extreme responses to immune checkpoint blockade following bipolar androgen therapy and enzalutamide in patients with metastatic castration resistant prostate cancer. Prostate.

[4] Baxevanis C.N., Fortis S.P., Ardavanis A., Perez S.A. (2020). Exploring Essential Issues for Improving Therapeutic Cancer Vaccine Trial Design. Cancers.

[5] van den Eertwegh A.J., Versluis J., van den Berg H.P., Santegoets S.J., van Moorselaar R.J., van der Sluis T.M., Gall H.E., Harding T.C., Jooss K., Lowy I. (2012). Combined immunotherapy with granulocyte-macrophage colony-stimulating factor-transduced allogeneic prostate cancer cells and ipilimumab in patients with metastatic castration-resistant prostate cancer: A phase 1 dose-escalation trial. Lancet Oncol..

[6] McNeel D.G., Eickhoff J.C., Wargowski E., Zahm C., Staab M.J., Straus J., Liu G. (2018). Concurrent, but not sequential, PD-1 blockade with a DNA vaccine elicits anti-tumor responses in patients with metastatic, castration-resistant prostate cancer. Oncotarget.

[7] McNeel D.G., Eickhoff J.C., Wargowski E., Johnson L.E., Kyriakopoulos C.E., Emamekhoo H., Lang J.M., Brennan M.J., Liu G. (2022). Phase 2 trial of T-cell activation using MVI-816 and pembrolizumab in patients with metastatic, castration-resistant prostate cancer (mCRPC). J. Immunother. Cancer.

[8] Exner R., Sachet M., Arnold T., Zinn-Zinnenburg M., Michlmayr A., Dubsky P., Bartsch R., Steger G., Gnant M., Bergmann M. (2016). Prognostic value of HMGB1 in early breast cancer patients under neoadjuvant chemotherapy. Cancer Med..

[9] Vandenabeele P., Vandecasteele K., Bachert C., Krysko O., Krysko D.V. (2016). Immunogenic Apoptotic Cell Death and Anticancer Immunity. Adv. Exp. Med. Biol..

[10] Sanchez C., Chan R., Bajgain P., Rambally S., Palapattu G., Mims M., Rooney C.M., Leen A.M., Brenner M.K., Vera J.F. (2013). Combining T-cell immunotherapy and anti-androgen therapy for prostate cancer. Prostate Cancer Prostatic Dis..

[11] Fay E.K., Graff J.N. (2020). Immunotherapy in Prostate Cancer. Cancers.

